# Idiopathic Calcinosis Cutis over Elbow in a 12-Year Old Child

**DOI:** 10.1155/2013/241891

**Published:** 2013-11-04

**Authors:** S. K. Venkatesh Gupta, Ramana Rao Balaga, Suman Kumar Banik

**Affiliations:** Department of Orthopaedics, Mamata Medical College/General Hospital, Khammam, Andhra Pradesh 507002, India

## Abstract

Calcinosis cutis is an uncommon disorder caused by an abnormal deposit of calcium phosphate in the skin in various parts of the body. Four main types of calcinosis cutis have been recognized according to etiology: associated with localized or widespread tissue changes or damage (*dystrophic calcification*), that associated with an abnormal calcium and phosphorus metabolism (*metastatic calcification*), not associated with any tissue damage or demonstrable metabolic disorder (*idiopathic calcification*), and *Iatrogenic.* Very few cases of idiopathic calcinosis cutis are reported in early childhood in the literature. We report one such case of idiopathic calcinosis cutis over elbow in a 12-year-old female child. Histological examinations of the lesions resected in this case reveal calcium deposits in the dermis, surrounded by foreign body giant cells. Idiopathic calcinosis cutis is a rare phenomenon and occurs in the absence of known tissue injury or systemic metabolic defect. It is important to delineate it from other calcification disorders for further plan of management. Medical therapy in calcinosis cutis is of limited benefit in pediatric age group and poses a challenging problem of postsurgical management.

## 1. Introduction 

Calcinosis cutis is a term used to describe a group of disorders in which calcium deposits form in the skin. Virchow initially described calcinosis cutis in 1855 [1]. Calcinosis cutis is classified into 4 major types according to etiology: dystrophic, metastatic, iatrogenic, and idiopathic [1]. Dystrophic calcinosis is calcification associated with infection, inflammatory processes, cutaneous neoplasm, or connective tissue diseases [2, 3]. Idiopathic calcinosis cutis is cutaneous calcification of unknown cause with normal serum calcium [4]. Subepidermal calcified nodule and tumoral calcinosis are idiopathic forms of calcification. Metastatic calcification results from elevated serum levels of calcium or phosphorus [5]. Iatrogenic and traumatic calcinosis are those types which are associated with medical procedures [5]. A few rare types have been variably classified as dystrophic or idiopathic [6]. These include calcinosis cutis circumscripta, calcinosis cutis universalis, tumoral calcinosis, and transplant-associated calcinosis cutis [6].

 Calcinosis cutis with Raynaud's phenomenon, oesophageal dysmotility, sclerodactyly, and telangiectasia is referred to as CREST syndrome [7–9]. The term “idiopathic calcinosis” is used when neither local tissue injury nor systemic metabolic disorder can be demonstrated [4]. Very few cases of idiopathic calcinosis cutis are reported in early childhood in the literature. We present a case of idiopathic calcinosis cutis over elbow joint in a 12-year-old female child.

## 2. Pathophysiology

In all cases of calcinosis cutis, insoluble compounds of calcium (hydroxyapatite crystals or amorphous calcium phosphate) are deposited within the skin due to local or systemic factors. Metabolic and physical factors are pivotal in the development of most cases of calcinosis. Ectopic calcification can occur in the setting of hypercalcemia and hyperphosphatemia. These elevated extracellular levels may result in increased intracellular levels, calcium-phosphate nucleation, and crystalline precipitation. Alternatively, damaged tissue may allow an influx of calcium ions leading to an elevated intracellular calcium level and subsequent crystalline precipitation. Tissue damage also may result in denatured proteins that preferentially bind phosphate. Calcium then reacts with bound phosphate ions leading to precipitation of calcium phosphate in the tissues [10].

Commonly, the skin and subcutaneous fat are involved but the deeper soft tissues may also be affected [10]. The calcified material may form palpable nodules, induce muscle atrophy, and predispose to the formation of contractures [11]. Local inflammation may occur, leading to ulceration and extrusion of calcified material.

## 3. Case Report

A 12-year-old female patient presented to our OPD with complaints of swelling over the posterior aspect of her right elbow of 45 days duration. Swelling was sudden in onset, initially of a size of a one-rupee coin and gradually progressed to about 5 × 4 × 2 cm at the time of presentation. Patient did not give any history of trauma/prick injury. There was no history of any kind of immobilization or massage treatment. No similar swellings elsewhere in the body. Patient complained of pain during lifting weights or when direct pressure is applied. 

**Figure 1:**
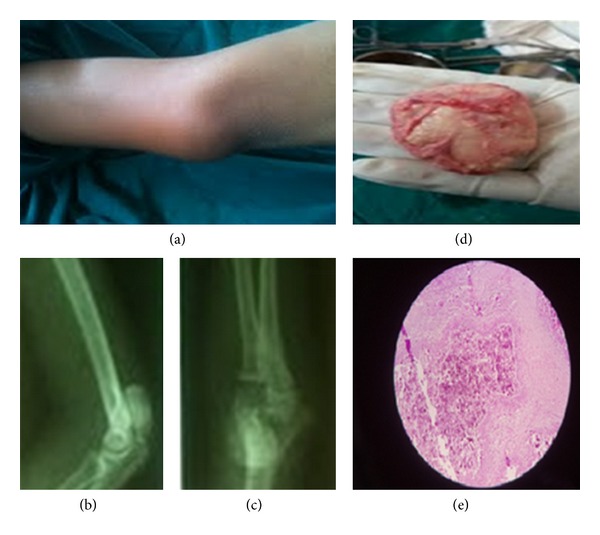
(a) Clinical picture of elbow with swelling and erythema; (b) and (c) X-ray of elbow AP and lateral views showing calcified mass over distal end of humerus; (d) gross morphology of excised specimen; (e) microscopic picture showing homogenous calcified mass with fibrous septa with giant cells and macrophages within.

On examination, swelling extended from the lower third of right arm to the elbow joint. It was globular in shape with the overlying skin being pinchable and erythematous. Swelling was firm in consistency with mild tenderness and no local rise of temperature. It is mobile in both horizontal and vertical directions suggestive of nonfixity to the underlying bone. All movements at elbow were unrestricted and pain free. There was no muscle wasting and sensations over the arm and forearm were intact without any distal neurovascular deficits (Figure 1).

### 3.1. Evaluation and Approach to Management

Serum calcium (10.2 mg/dL), serum phosphorus (3.0 mg/dL), serum Alkaline Phosphatase (127 IU/L), parathyroid hormone levels, creatinine kinase, aldolase levels, ANA, Vitamin D levels, 24 hours urinary calcium, and inorganic phosphate were within the normal limits. An ultrasonographic scan revealed a well-defined and calcified intramuscular lesion measuring 4.1 × 3.4 cm in the lower end of triceps muscle, with no fixity to humerus. Radiograph showed well-defined calcified mass over the posterior aspect of the distal end of the humerus extending up to the elbow joint.

**Figure 2:**
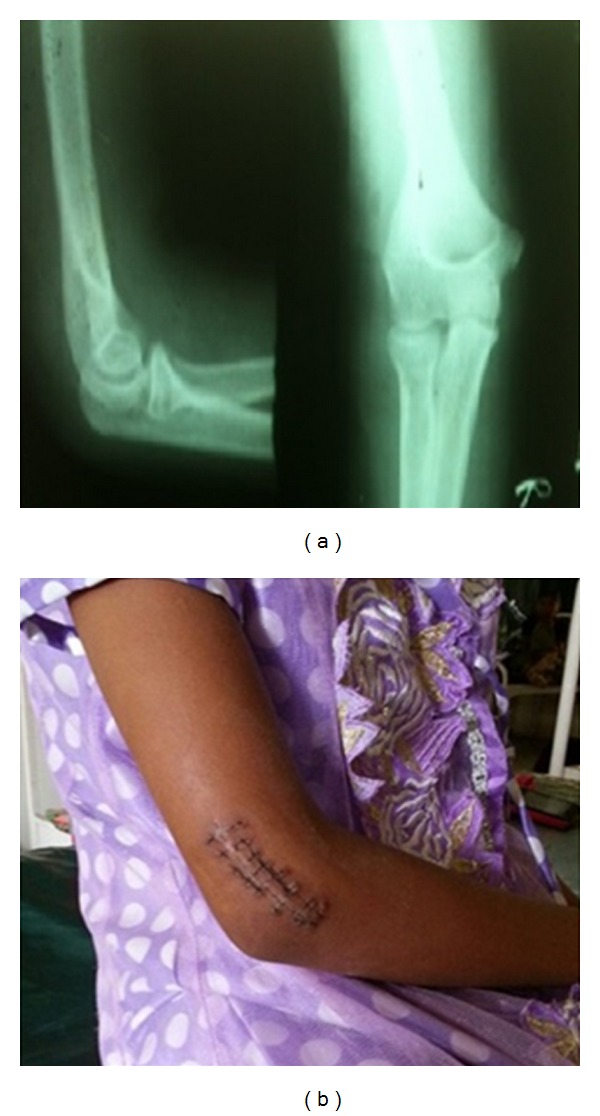
(a) Postoperative radiograph at 1-year follow up showing no evidence of recurrence. (b) Postoperative scar photograph after excision biopsy.

With the above clinical scenario, we opted for excision biopsy that revealed a single encapsulated grey white mass of tissue measuring 4 × 3.5 × 2 cm overlying the posterior aspect of humerus in the triceps muscular plane (Figure 2).

Microscopic description showed large homogenous masses of calcified material separated by thick fibrous bands of septa. Numerous giant cells and macrophages were observed in the septa confirming it to be calcinosis cutis.

Patient was followed up to a period of one year, where there were no signs of recurrence.

## 4. Discussion

Calcinosis cutis is a term used to describe a group of disorders in which aberrant calcium deposits form in the skin. Various types of calcinosis cutis have been previously described [2, 3, 5–9]. It is very important to diagnose the exact type of calcinosis so that treatment can be accurately rendered for effective management. In the present case scenario, all the investigations to evaluate abnormal calcium metabolism: Serum calcium, Serum phosphorus, and Serum ALP were within normal limits and there was no history of trauma/injury/constitutional symptoms; hence, it was diagnosed as idiopathic calcinosis cutis, where in the etiology it is not known and its pathogenesis is not clearly understood.

Idiopathic calcinosis cutis is a rare phenomenon and poses a special challenge in view of recurrence, particularly in children. Although excision of a large calcified mass is opined, its recurrence is not uncommon. Medical line of treatment after excision is a recommended protocol in such cases. Intralesional corticosteroids may be beneficial. Probenecid and colchicine have been useful in some patients. Magnesium or aluminium antacids may be effective phosphate binders in patients with hyperphosphatemia [12]. However, their use in renal insufficiency may result in toxicity. Sodium etidronate and bisphosphonates may be helpful in some individuals by reducing the bone turnover and inhibiting the growth of ectopic hydroxyapatite crystals. However, treatment is prolonged and paradoxical hyperphosphatemia may be a result. Warfarin has shown benefit in some cases. There have been variably beneficial effects with the use of calcium channel blocker, Diltiazem over the period of last five years. The therapeutic effect of this is believed to be antagonism of calcium-sodium ion pump [13, 14]. The above medical management, though beneficial in some cases, is not recommended for children as the risks outweigh the benefits, thereby posing a challenge to the medical fraternity in the treatment of idiopathic calcinosis cutis. Probenecid and colchicine are contraindicated in the pediatric age group with major side effects being hypersensitive reactions and defective spermatogenesis. Calcium channel blockers pose the risk of liver dysfunction, hypersensitivity reactions, tachycardia, and pedal edema in children. Warfarin is known for its potential risk of occult bleed/haemorrhage. 

Unless the exact pathogenesis or dystrophic variant of calcinosis cutis is determined, medical line of prevention after surgical excision has more potential complications in children. In the present case, there was no underlying, evident defective calcium metabolism and hence we opted for dietary regulation of preventing hypercalcemia than pharmacological control. We did not encounter any recurrence at one-year of follow up.

## 5. Conclusion

After clinical diagnosis of calcinosis cutis, a laboratory workup to rule out abnormalities of calcium and phosphorus metabolism, malignant processes, collagen vascular diseases, renal insufficiency, excessive milk ingestion, and vitamin D poisoning must be carried out to detect the underlying cause of the disease. In conclusion, we recommend excision of the lesion as it does not only provide successful resolution without recurrence but also establishes the diagnosis.
